# Some aspects of radical chemistry in the assembly of complex molecular architectures

**DOI:** 10.3762/bjoc.9.61

**Published:** 2013-03-18

**Authors:** Béatrice Quiclet-Sire, Samir Z Zard

**Affiliations:** 1 Laboratoire de Synthèse Organique, CNRS UMR 7652, Ecole Polytechnique, 91128 Palaiseau, France

**Keywords:** amidyls, iminyls, radical allylation, radical vinylation, xanthate transfer

## Abstract

This review article describes briefly some of the radical processes developed in the authors’ laboratory as they pertain to the concise assembly of complex molecular scaffolds. The emphasis is placed on the use of nitrogen-centred radicals, on the degenerate addition–transfer of xanthates, especially on its potential for intermolecular carbon–carbon bond formation, and on the generation and capture of radicals through electron transfer processes.

## Introduction

Natural products exhibit an astonishing diversity of molecular architectures and structural complexity. This has spurred the development of numerous synthetic strategies for the rapid assembly of intricate carbon frameworks. In this context, reactions allowing the concomitant or sequential formation of multiple new bonds acquire a special importance [1]. Radicals, in particular, have proved to be especially apt for such a task, and numerous cascade or domino sequences have been described over the past three decades [2–5]. Radicals offer many of the properties desired by synthetic organic chemists, as compared to ionic or organometallic reactions: generally mild and neutral experimental conditions; lower sensitivity to steric hindrance; lower susceptibility to the solvent effects; lesser tendency for rearrangements and β-elimination; and a selectivity that is often complementary to that of ionic or organometallic reactions, making some protection steps superfluous. Radicals are ambiphilic species that can react with both electron-poor and electron-rich substrates, but the rates can differ by several orders of magnitude. The result is a broad spectrum of reactivity that is, paradoxically, often accompanied by a remarkable selectivity.

A major part of our research effort is directed towards the design and development of new radical processes. These include methods for the generation and capture of nitrogen-centred radicals, the degenerative transfer of xanthates and related thiocarbonylthio derivatives, chain processes based on the chemistry of sulfonyl radicals, and electron transfer from metallic nickel to halides and oxime esters. These new reactions can be readily harnessed for the rapid creation of complex molecular structures. The present brief overview aims at giving an idea of the synthetic possibilities.

## Review

### Nitrogen-centred radicals

Nitrogen radicals have received little attention from synthetic organic chemists, in contrast to carbon radicals. Yet their potential for the creation of C–N bonds and for the synthesis of alkaloids is enormous [6]. One possible explanation is the lack, hitherto, of mild yet general methods for the generation of the various types of nitrogen radicals: aminyls, aminiums, iminyls, amidyls, carbamyls, ureidyls, etc.

**Scheme 1:**
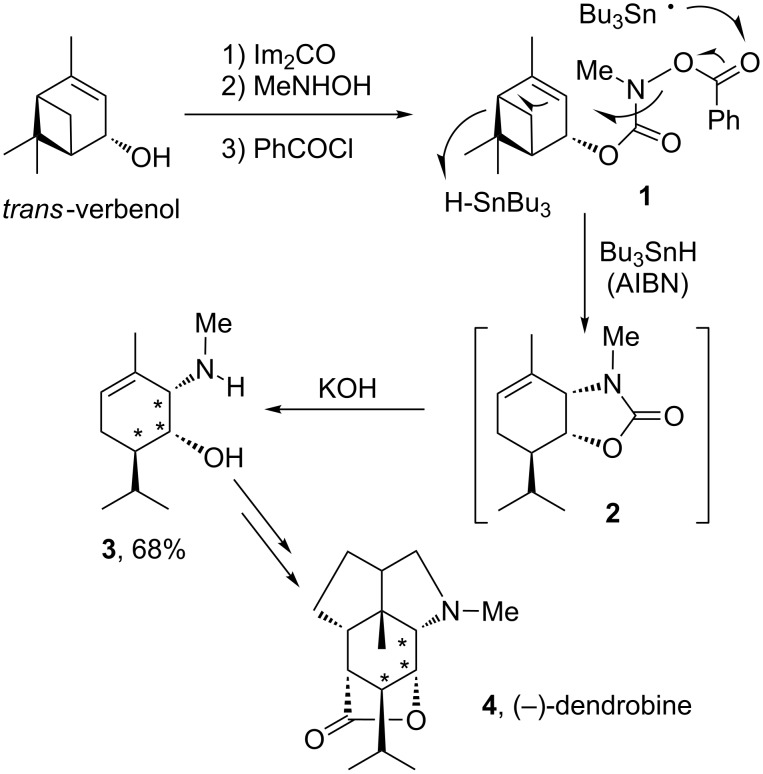
Key radical step in the total synthesis of (–)-dendrobine.

We discovered some years ago that benzoates of hydroxamic acids, oximes and related derivatives reacted nicely with stannyl radicals to furnish the corresponding nitrogen radicals under mild conditions [7]. These could be readily incorporated into various radical sequences leading to complex nitrogen-containing scaffolds. One illustration is provided by the central transformation in the total synthesis of (–)-dendrobine (**4**), where the carbamyl radical cyclisation is followed by rupture of the cyclobutane ring [8–9]. This operation, displayed in Scheme 1, starts with benzoate **1** and results in the formation of the carbon–nitrogen bond with the correct stereochemistry and the introduction of the pendant isopropyl group present in the target. The intermediate cyclic carbamate **2** is not isolated but cleaved into aminoalcohol **3** to simplify purification. The conversion of aminoalcohol **3** into (–)-dendrobine (**4**), hinges on the use of the fabulous Pauson–Khand reaction [10–11] to introduce simultaneously the two adjacent five-membered rings.

**Scheme 2:**
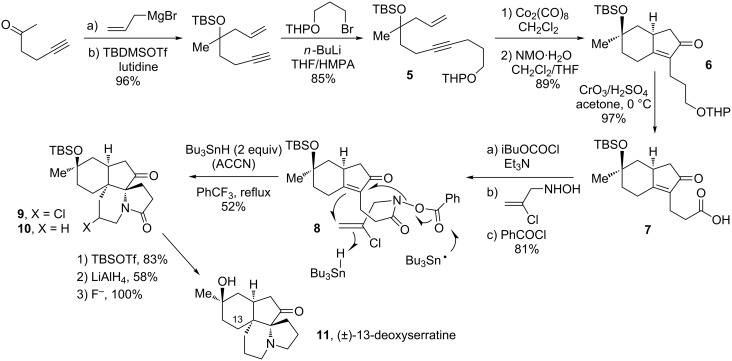
Radical cascade in the total synthesis of (±)-13-deoxyserratine (ACCN = 1,1'-azobis(cyclohexanecarbonitrile)).

Outlined in Scheme 2 is a very short total synthesis of (±)-13-deoxyserratine (**11**) [12–13]. The first two of the four rings in **11** are again created through the powerful Pauson–Khand reaction starting from trivial enyne **5** and oxidation of the protected alcohol in the side-chain of cyclopentenone **6** directly into carboxylic acid **7** by using Jones’ reagent. The second key step, namely the conversion of benzoate **8** into tetracycle **10**, involves the concurrent formation of two rings and two adjacent quaternary centres. The chlorine atom in **8** is deliberately introduced in order to direct the second cyclisation towards the 6-*endo* mode. The chloride in intermediate **9** is now aliphatic and not vinylic as in precursor **8**; it is therefore more susceptible to attack by stannyl radicals and the addition of an extra equivalent of tributylstannane ensures its in situ reductive removal. This domino radical cyclisation represents, in fact, a general strategy for the construction of indolizidines and pyrrolizidines, which constitute the core structure of numerous alkaloids. For pyrrolizidines, one needs simply to allow the second cyclisation to proceed in a 5-*exo* fashion by starting with an un-chlorinated substrate.

**Scheme 3:**
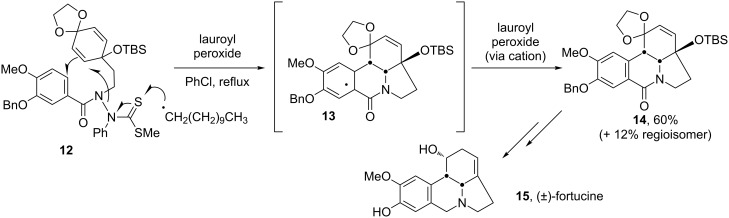
Formation of the complete skeleton of (±)-fortucine.

The total synthesis of fortucine (**15**), also hinges on the sequential fashioning of an indolizidine-type skeleton, with the second cyclisation taking place on an aromatic ring (Scheme 3) [14–15]. The formation of the amidyl radical in this case calls for a different precursor and does not involve a stannane reagent. The sequence is triggered by the attack of undecyl radicals on thiosemicarbazone **12**. Undecyl radicals arise from the thermal homolysis of lauroyl peroxide and decarboxylation. The lauroyl peroxide must be used in stoichiometric amounts, for it is required to oxidise the intermediate cyclohexadienyl radical **13** into its corresponding cation and thence into intermediate **14** by rapid loss of a proton.

**Scheme 4:**
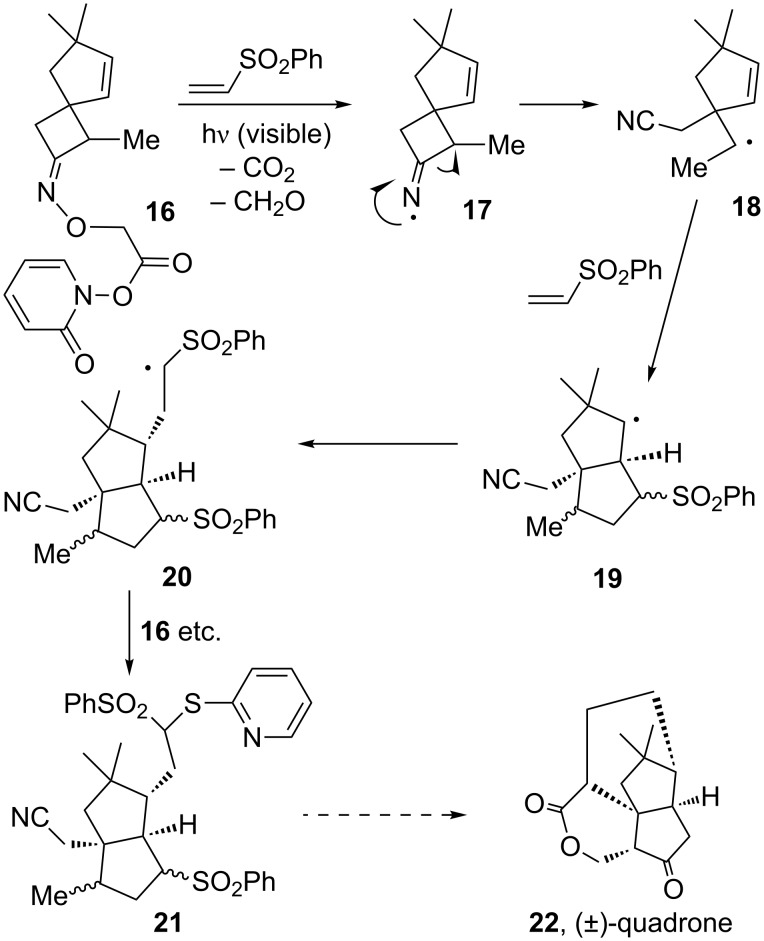
Model radical sequence for the synthesis of quadrone.

A faster access to complexity is obtained when intermolecular steps are also involved. This increases considerably the convergence of the synthetic scheme. One such instance is pictured in Scheme 4, whereby cyclobutyliminyl radical **17**, generated from **16** by a modified Barton decarboxylation [16], undergoes a regioselective scission into secondary radical **18**, followed by an initial intermolecular addition to phenyl vinyl sulfone and closure to form the cyclopentane ring in **19**, and then by a second intermolecular addition to phenyl vinyl sulfone to furnish radical **20,** which finally evolves into the observed product **21** by transfer of the pyridylthiyl group from the starting Barton ester **16**. Even though the yield is still on the order of 40% and needs to be optimised, this sequence introduces all the carbons needed for a projected synthesis of (±)-quadrone (**22**) [17].

**Scheme 5:**
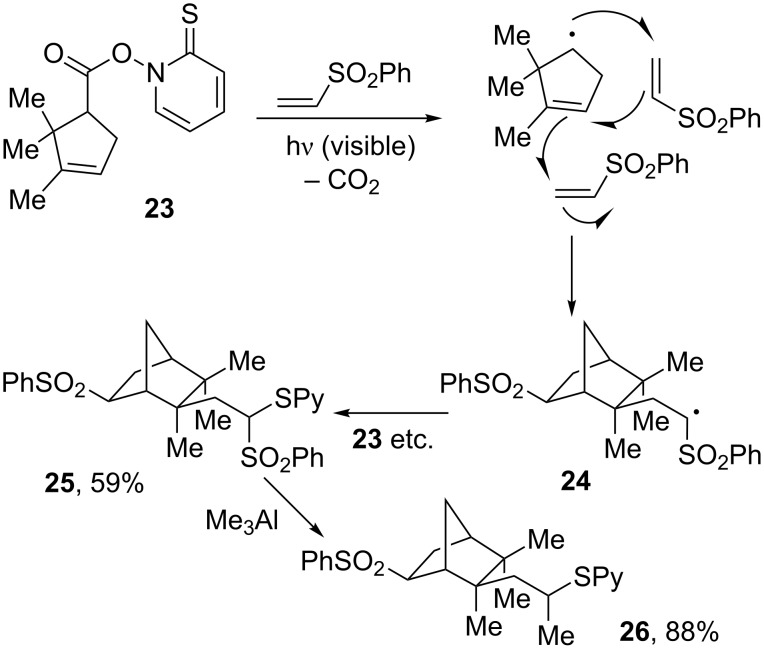
Radical cascade using the Barton decarboxylation.

This remarkable radical cascade is in fact inspired by an earlier transformation shown in Scheme 5 and starting from simple carboxylic acid derivative **23** [18]. As in the previous sequence, radical **24**, generated following the second addition to the vinyl sulfone, is electrophilic in character and does not add further; it simply propagates the chain by reacting with the starting thiohydroxamate **23**. The complex bicyclic structure **25** is thus made in just one step. In both of these examples, three C–C bonds and one C–S bond are formed one after the other, resulting in a considerable increase in complexity. Interestingly, and not unexpectedly, the two sulfones in structures **21** and **25** have very different reactivities. For instance, exposure of bis-sulfone **25** to trimethylaluminium causes the regioselective replacement of the terminal sulfone with a methyl group to give compound **26** in good yield. The Barton decarboxylation reaction is an exceptionally powerful method that deserves without doubt a much greater attention from synthetic organic chemists.

### The degenerative transfer of xanthates

**Scheme 6:**
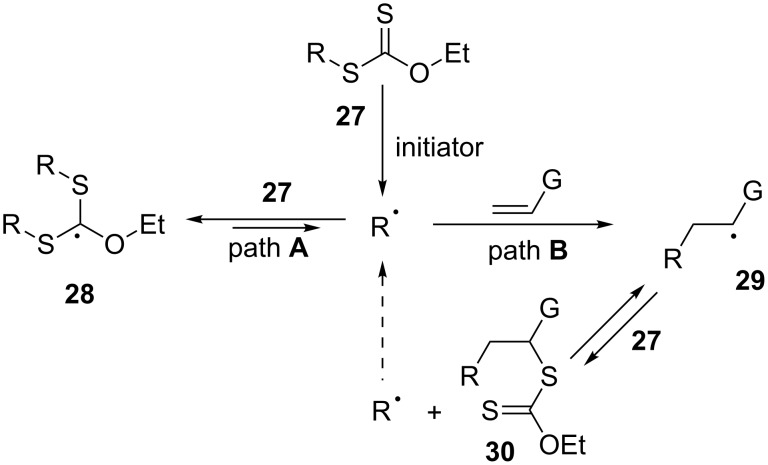
Simplified mechanism for the xanthate addition to alkenes.

A longstanding challenge in organic synthesis has been the intermolecular creation of new carbon–carbon bonds starting with simple unactivated alkenes. The cross-metathesis constitutes at the present time one of the better solutions to this problem [19]. Another solution is the tin-free degenerative radical transfer of xanthates and related derivatives we discovered a quarter of a century ago [20]. The simplified mechanism for the addition to an alkene is depicted in Scheme 6.

The many subtle aspects embodied in the mechanism will not be discussed for lack of space, but the interested reader is directed to a recent review for a more complete description [21]. Experimentally, the procedures are simple and safe, and the reagents are cheap and readily available. In the present context, two properties are especially noteworthy:

(a) The reaction of radical R· with its xanthate precursor **27** to give adduct **28** is fast but degenerate (path **A**). Radical R· is therefore continuously regenerated and acquires an extended effective lifetime. It can thus be readily captured intra- or intermolecularly, even by unactivated olefinic traps, to give finally adduct **30** (path **B**). More generally, relatively slow radical processes (additions, cyclisations, fragmentations, etc.) can be accomplished, without need for high dilution or syringe-pump techniques. This property expands considerably the scope by allowing transformations not feasible with other radical methods. The main limitation is that the initial radical R· has to be more stable than adduct radical **29** in order to bias the equilibrium in favour of product **30** and avoid the formation of oligomers by further additions of radical **29** to the alkene. Radicals R· stabilised by electron-withdrawing groups (nitrile, ketones, esters, pyridines, tetrazoles, etc.) are particularly suitable. Benzyl radicals are not reactive enough towards unactivated alkenes; they tend to accumulate in the medium and ultimately dimerise.

(b) The addition product, **30**, being itself a xanthate, allows the implementation of a second radical sequence, leading in turn to yet another xanthate. Alternatively, the xanthate group can be exploited as an entry into the extremely rich "ionic" chemistry of sulfur. Thus, a plethora of transformations can be easily marshalled to introduce further diversity and complexity into the structures.

By allowing intermolecular radical additions on unactivated alkenes, the xanthate transfer process opens infinite possibilities for bringing together various functional groups, which can then be made to react together. The functional groups can be present on the xanthate and/or the alkene partners. Over 2000 additions have so far been performed using more than 100 different xanthates. The few examples presented hereafter will hopefully offer a glimpse of the potential for accessing complexity.

**Scheme 7:**
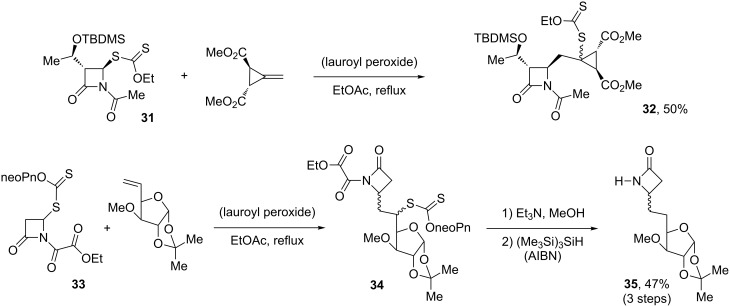
Synthesis of β-lactam derivatives.

The neutral, mild experimental conditions translate into a broad tolerance for sensitive functionality. This aspect is encapsulated in the two addition reactions presented in Scheme 7 [22]. β-Lactam xanthates such as **31** and **33** can be readily added without harm to the fragile azetidinone motif and, if desired, the xanthate group may be reduced off by a number of methods, the mildest perhaps relying on tris(trimethylsilyl)silane as the reducing agent [23]. Its use is illustrated by the synthesis of the β-lactam-sugar conjugate **35**, which also highlights the possibility of cleanly removing an oxalyl group from adduct **34** without destruction of the azetidinone [24]. The synthesis of complex compounds such **32** and **35** would be very tedious by more traditional routes.

**Scheme 8:**
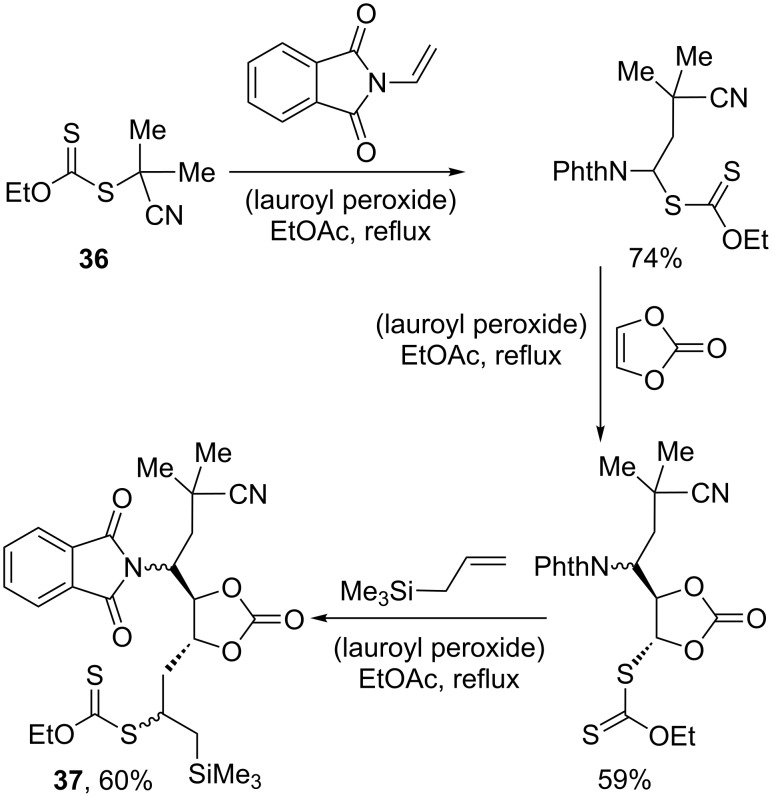
Sequential additions to three different alkenes (PhthN = phthalimido).

The possibility of iterating the radical addition allows for a convergent and highly modular assembly of complex scaffolds. The three successive additions outlined in Scheme 8 and leading to **37** illustrate nicely this approach [25]. One xanthate, **36**, and three different alkenes are stitched together using the same experimental conditions. The order of the additions is, however, important. One of the requirements, implicit in the general mechanism pictured in Scheme 6, is that the initial radical R· has to be more stable than adduct radical **29**, neglecting polar effects in a first approximation (again, [21] may be consulted for a more thorough discussion). A detailed inspection of the various radicals implicated in the sequence in Scheme 8 would reveal that this condition has indeed been respected.

**Scheme 9:**
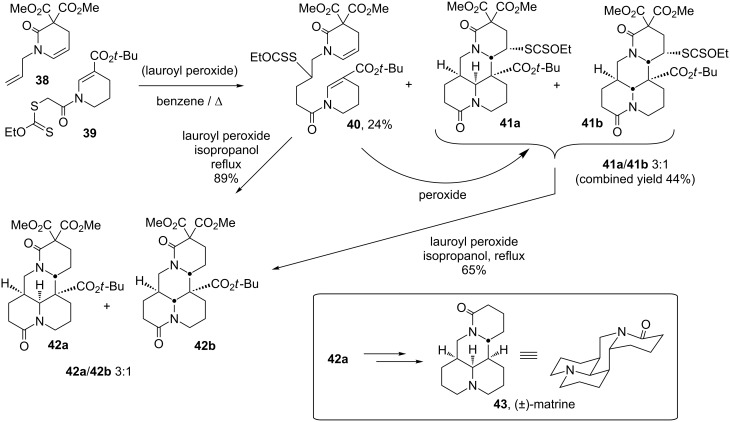
Key cascade in the total synthesis of (±)-matrine (**43**).

Nevertheless, numerous variations in such sequential radical additions can be readily conceived. This is demonstrated by the key step in the total synthesis of matrine (**43**), which starts with readily available alkene **38** and xanthate **39**, and implies one intermolecular addition followed by two successive cyclisations [26]. Two isomeric tetracyclic compounds **41a** and **41b** as well as simple addition product **40** are thus obtained in good combined yield (Scheme 9). The latter may be converted into the same mixture of **41a** and **41b** by further treatment with peroxide. In practice, however, it is more convenient to subject adduct **40** separately to a reductive double cyclisation to give the epimeric mixture of **42a** and **42b**, by using isopropanol both as the solvent and source of hydrogen atoms. The mixture of **42a** and **42b** is separated at this stage and the major isomer processed into matrine (**43**) (the minor isomer has the relative stereochemistry of allo-matrine, also a natural product).

**Scheme 10:**
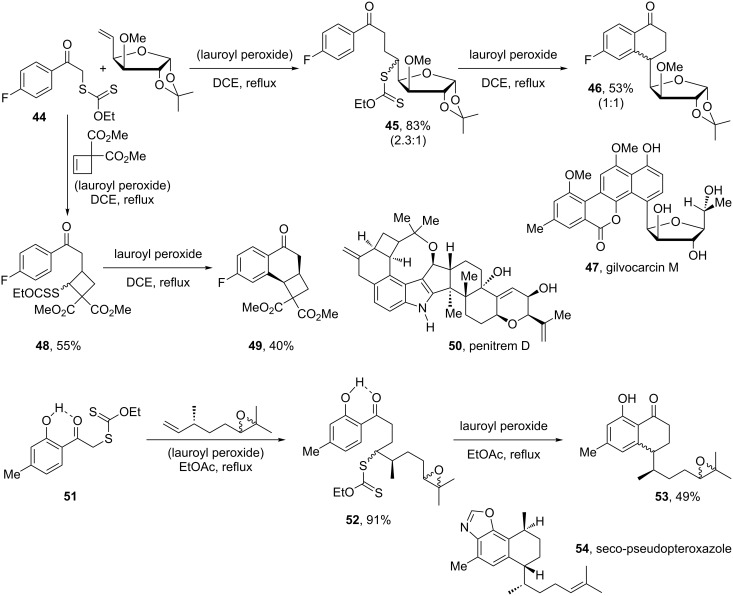
Synthesis of complex tetralones.

One important property of the xanthate addition–transfer process is that cross-over into a cationic manifold is possible if one of the intermediate radicals can be easily oxidised by the peroxide. The peroxide then acts as both the initiator and the stoichiometric oxidant. This is especially useful in the case of cyclisations onto aromatic or heteroaromatic rings (cf. synthesis of fortucine (**15**) in Scheme 3 above). The intermediate cyclohexadienyl radicals (analogous to radical **13**) cannot propagate the chain but are easily oxidised by the peroxide, which has to be used in stoichiometric amounts. Numerous aromatic derivatives can thus be very easily obtained. In Scheme 10, the synthesis of various tetralones is displayed. The addition of xanthate **44** to two different alkenes furnishes adducts **45** and **48**, and these can in turn be cyclised into tetralones **46** and **49**, respectively. The former represents a model study for the total synthesis of gilvocarcin M (**47**), a natural *C*-glycoside [27]; while the latter illustrates the possibility of constructing a cyclobutane-containing tricyclic motif related to the one found in penitrem D, **50** [28]. The two-step formation of tetralone **53**, starting from xanthate **51** and proceeding via adduct **52**, underscores the tolerance of the process for the presence of an epoxide and, at least in this case, of a free phenol [29]. This sequence represents an attractive potential route to seco-pseudopteroxazole, **54**, and to other congeners in this family.

**Scheme 11:**
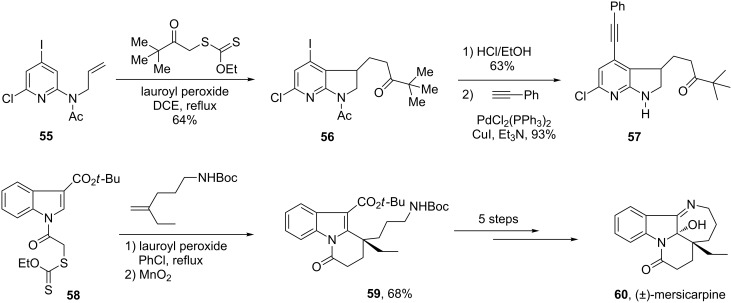
Synthesis of functionalised azaindoline and indole derivatives.

In many cases, both the intermolecular addition and the ring closure can be performed in the same flask, as shown by the direct synthesis of azaindoline **56** from alkene **55** (Scheme 11) [30]. Azaindoline **56** contains at least four orthogonal sites for diversification, in addition to the obvious possibility of varying the starting xanthate. For instance, a regioselective Sonogashira coupling leading to compound **57** may be performed without affecting the less reactive chlorine substituent. The annelation commencing with xanthate **58** and furnishing indole derivative **59**, an advanced intermediate in the formal synthesis of mersicarpine (**60**), is another illustration [31]. The aromatisation process in this example was incomplete under the usual conditions and required further treatment with manganese dioxide.

**Scheme 12:**
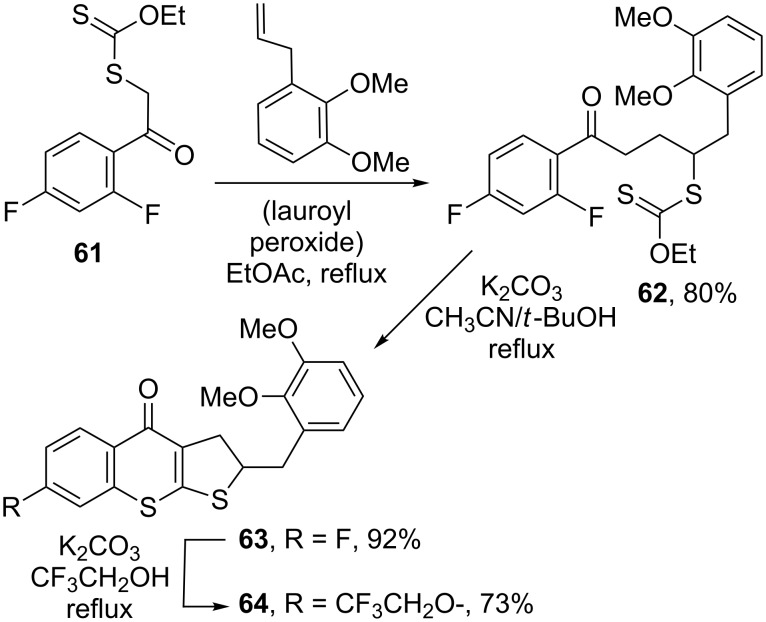
Synthesis of thiochromanones.

The possibility of associating the radical chemistry of xanthates with various ionic reactions represents another powerful strategy for creating complexity from simple starting materials. In this approach, the radical sequence brings together the functional groups necessary for the ionic transformation. One example is the sequence that follows the radical addition of xanthate **61** to an alkene depicted in Scheme 12. Exposure of adduct **62** to the action of potassium carbonate, in a mixture of acetonitrile and *tert*-butanol under reflux, leads to tricyclic thiochromanone **63** in high yield [32]. This transformation involves attack of the ketone enolate on the thiocarbonyl group of the nearby xanthate, followed by substitution of the fluorine by the sulfide anion thus produced, and loss of a molecule of ethanol. Such, hitherto rare, structures can now be accessed in two easy steps that could in principle be performed in one pot. Variety is readily obtained by merely modifying the alkene or by taking advantage of the presence of the remaining fluorine to introduce different substituents, as shown by the simple formation of trifluoroethoxy derivative **64**.

**Scheme 13:**
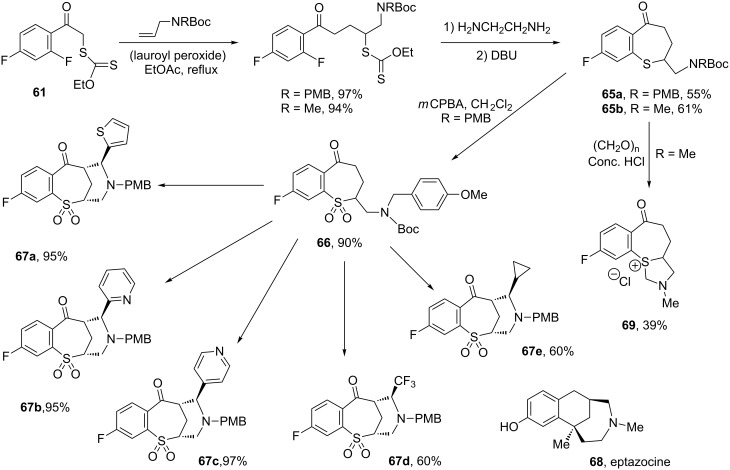
Synthesis of complex benzothiepinones. Conditions: 1) CF3COOH; 2) RCHO / AcOH (PMB = *p*-methoxybenzyl).

The adducts derived from xanthate **61** can be used in yet another way. Gentle aminolysis of the xanthate frees a thiol, which, under more basic conditions, displaces the *ortho*-fluorine to afford a benzothiepinone (Scheme 13) [32]. In the particular case of **65a**, oxidation to the corresponding sulfone **66** followed by a Mannich reaction with various aldehydes leads to a plethora of complex polycyclic structures **67a**–**e**, which may be viewed as analogues of eptazocine **68** [32]. Oxidation to the sulfone is necessary: when the Mannich reaction of benzothiepinone **65b** with formaldehyde was attempted, the reaction resulted in the formation of novel tricyclic sulfonium **69** in modest yield [32].

**Scheme 14:**
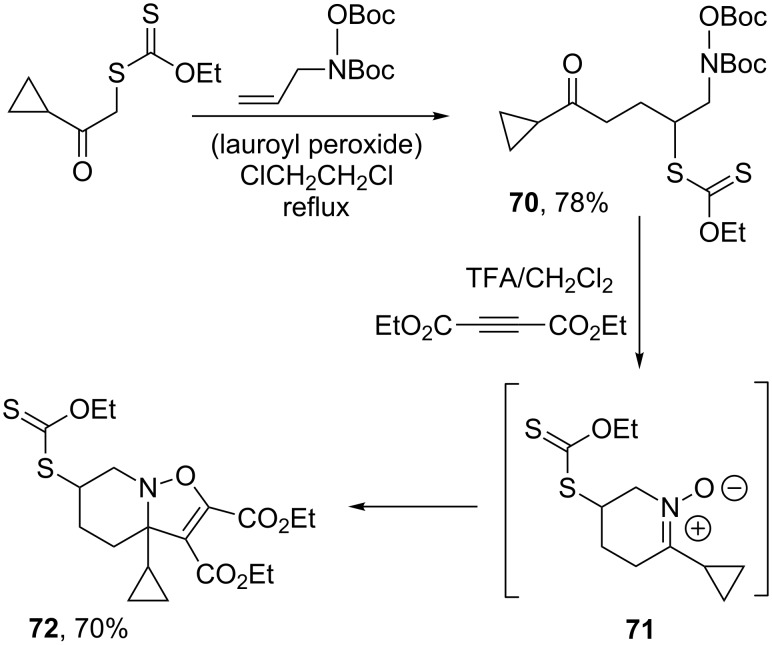
Formation and capture of a cyclic nitrone.

It is also possible to profit from the ability of xanthates to mediate additions to olefins containing hydroxylamine and hydrazine substituents in order to construct unusual heterocycles [33–34]. One typical illustration is presented in Scheme 14, where unmasking of the hydroxylamine in adduct **70** gives rise to cyclic nitrone **71**, which can then be intercepted by a dipolarophile placed in the medium, as shown by the ready formation of bicyclic compound **72** [33].

**Scheme 15:**
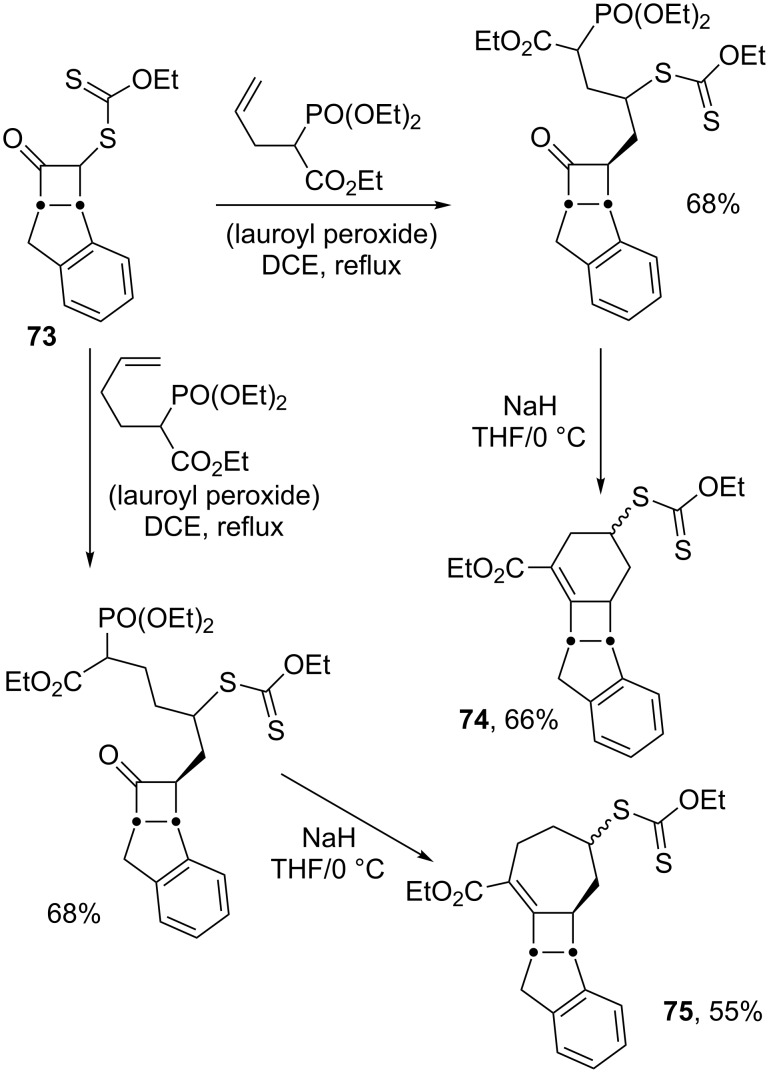
Synthesis of bicyclic cyclobutane motifs.

The use of a phosphonate-containing alkene allows the assembly of numerous polycyclic structures by combining the radical addition of a ketone-bearing xanthate with an intramolecular Horner–Wadsworth–Emmons condensation. This strategy is highlighted in Scheme 15 by the synthesis of bicyclic cyclobutane derivatives **74** and **75** starting from 2-xanthyl cyclobutanone **73** [35]. Thus, depending on the distance between the terminal alkene and the phosphonate, a six- or a seven-membered ring may be fused onto the cyclobutane nucleus.

**Scheme 16:**
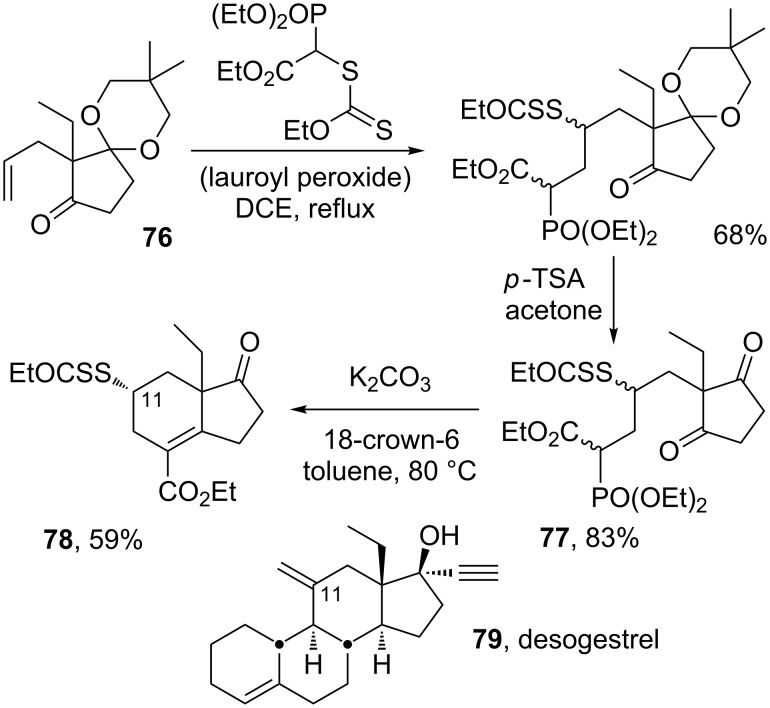
Construction of the CD rings of steroids.

This combination represents in fact a highly flexible route to polycyclic derivatives, since it is open to numerous variations. For instance, instead of having the phosphonate group attached to the alkene, it can be part of the xanthate component. Such a modification can be used to build the CD ring system found in the highly potent steroid contraceptive desogestrel (**79**), as outlined in Scheme 16 [36]. Thus, radical addition to alkene **76** furnishes intermediate **77**, after deprotection of the second ketone group. Exposure of the latter compound to base results in the formation of bicycle **78** as one diastereomer. While the condensation could in principle take place with either of the two ketones, it occurs in fact selectively with the ketone leading to the xanthate in the equatorial position in order to avoid a 1,3-diaxial interaction with the angular ethyl group. It is worth noting that the xanthate group conveniently occupies the 11-position (steroid numbering) and therefore allows the ready subsequent introduction of various substituents on this important position. In this quite general route to cyclohexenes, the requisite 2-allyl ketones are readily available by alkylation but also, and more importantly, by the exceedingly potent Claisen rearrangement [37], with the attending advantages of stereocontrol and chirality transfer.

**Scheme 17:**
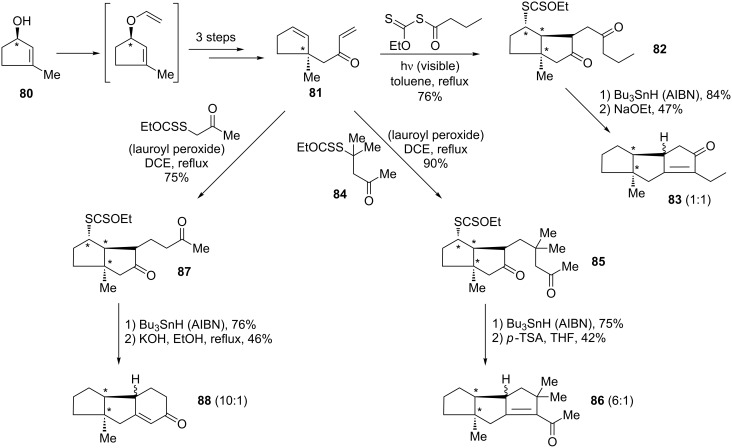
Rapid assembly of polyquinanes.

Another powerful approach to polycyclic structures is through association with Robinson-type annelations [38]. The synthesis of the precursors also exploits the Claisen rearrangement, as shown by the preparation of enone **81** from allylic alcohol **80** in Scheme 17. By varying the xanthate partner, different substitution patterns and rings may then be introduced. Two examples, **83** and **86**, in Scheme 17 illustrate the formation of triquinanes via bicyclic intermediate xanthates **82** and **85**. The former involves the addition of a butanoyl radical derived from the corresponding *S*-butanoyl xanthate, while the latter results from the addition of β-xanthyl ketone **84**. The use of an α-xanthyl ketone gives rise ultimately to a fused 6-membered ring, as shown by the formation of tricyclic product **88** from diquinane intermediate **87** [38]. In all of these transformations, the chiral information residing in the starting allylic alcohol **80** is transmitted, through the Claisen rearrangement, to various other centres (the ratios in Scheme 17 and following schemes refer to ratios of diastereoisomers). Very recently, this strategy was applied to a formal synthesis of (±)-hirsutic acid [39].

**Scheme 18:**
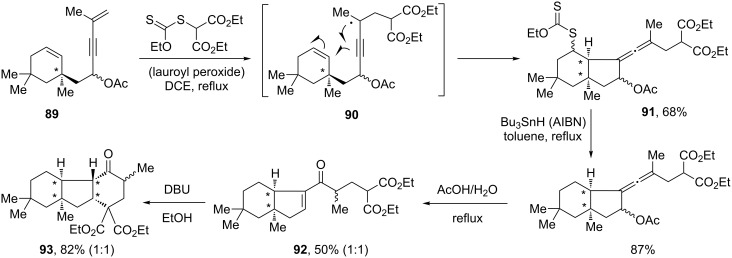
Formation of a polycyclic structure via an allene intermediate.

Access to polycyclic structures can be accomplished by cyclisation of propargyl radicals. Alkynes or allenes can be obtained, depending on the disposition of the internal alkene with respect to the delocalised radical [40–41]. In the sequence displayed in Scheme 18, the addition–cyclisation of a malonyl radical to enyne **89** furnishes allenyl acetate **91** by cyclisation of propargyl radical **90** [41]. Compound **91** readily undergoes reductive dexanthylation and solvolysis into enone **92**, and internal Michael addition to give tricyclic structure **93**. In this sequence too, the chirality present in the starting material **89** is initially derived from an allylic alcohol by the Claisen rearrangement and is then transmitted to the other centres.

**Scheme 19:**
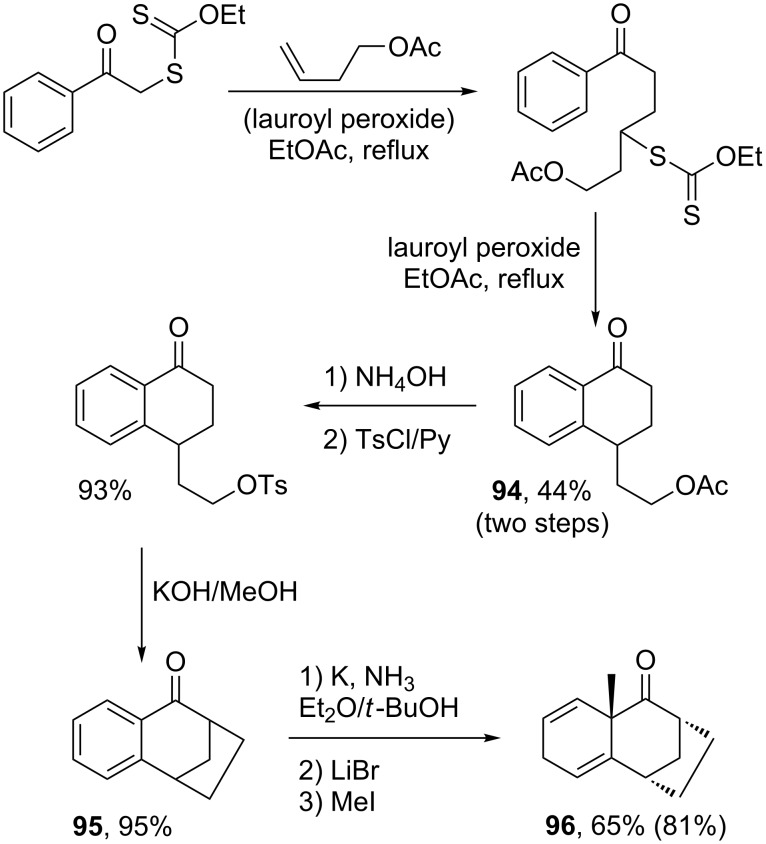
A polycyclic structure via the alkylative Birch reduction.

Another powerful reaction that can be associated with the radical chemistry of xanthates is the Birch reduction [42]. The radical process is employed to create the substituted aromatic motif, which is then reduced by the dissolving metal. Scheme 19 contains one such transformation, where the intermolecular radical addition is followed by ring closure to give tetralone **94**, which is easily converted into tricyclic derivative **95**. An alkylative Birch reduction finally furnishes **96** containing an angular methyl group [43]. This strategy lends itself in principle to numerous modifications, providing access to various ring combinations and substitution patterns.

**Scheme 20:**
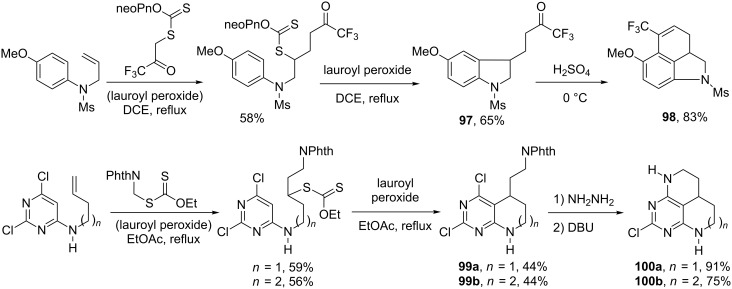
Synthesis of polycyclic pyrimidines and indoline structures.

The facility of introducing polar groups such as ketones and esters through the intermolecular radical-addition step allows the association of the radical ring-closure to aromatic and heteroaromatic derivatives with ionic cyclisation processes. Two such sequences are pictured in Scheme 20. The first combines the radical addition–cyclisation leading to indoline **97** with an intramolecular Friedel–Crafts reaction to afford a tricyclic derivative **98** substituted by a trifluoromethyl group [44]. The second exploits the presence of both a protected primary amine and an easily substitutable chlorine on the pyrimidine ring in **99a**,**b** to afford two interesting tricylic compounds, one of which, **100a**, is symmetrical, and the other, **100b**, contains a seven-membered ring [45].

**Scheme 21:**
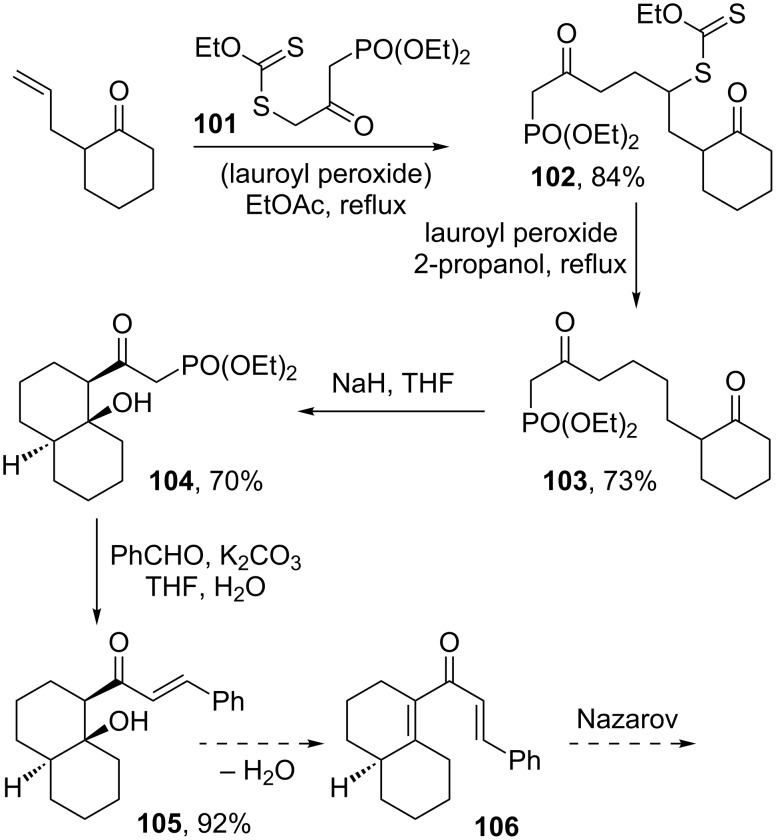
Construction of a *trans*-decalin derivative.

One path to complexity is through the use of conjunctive reagents, which can mediate an orthogonal two-directional formation of C–C bonds. In the context of xanthates, two such reagents have been studied. The first is ketophosphonyl xanthate **101**, where the intermolecular radical addition on one side of the ketone can be followed by a Horner–Wadsworth–Emmons (HWE) condensation on the other side [46]. In the transformation depicted in Scheme 21, compound **103**, obtained by the reductive dexanthylation of adduct **102** derived from 2-allylcyclohexanone, does not undergo an intramolecular HWE condensation to give a cyclooctene derivative upon treatment with sodium hydride. The formation of the cyclooctene ring is not very favourable and cannot compete with the simple aldol process leading to *trans*-decalin **104**. This compound readily undergoes HWE condensation with an external aldehyde, such as benzaldehyde. It is worth noting that β-elimination of water from the resulting product **105** would lead to dienone **106**, an interesting substrate for the Nazarov reaction which, in this case, would fuse a cyclopentenone ring on the structure.

**Scheme 22:**
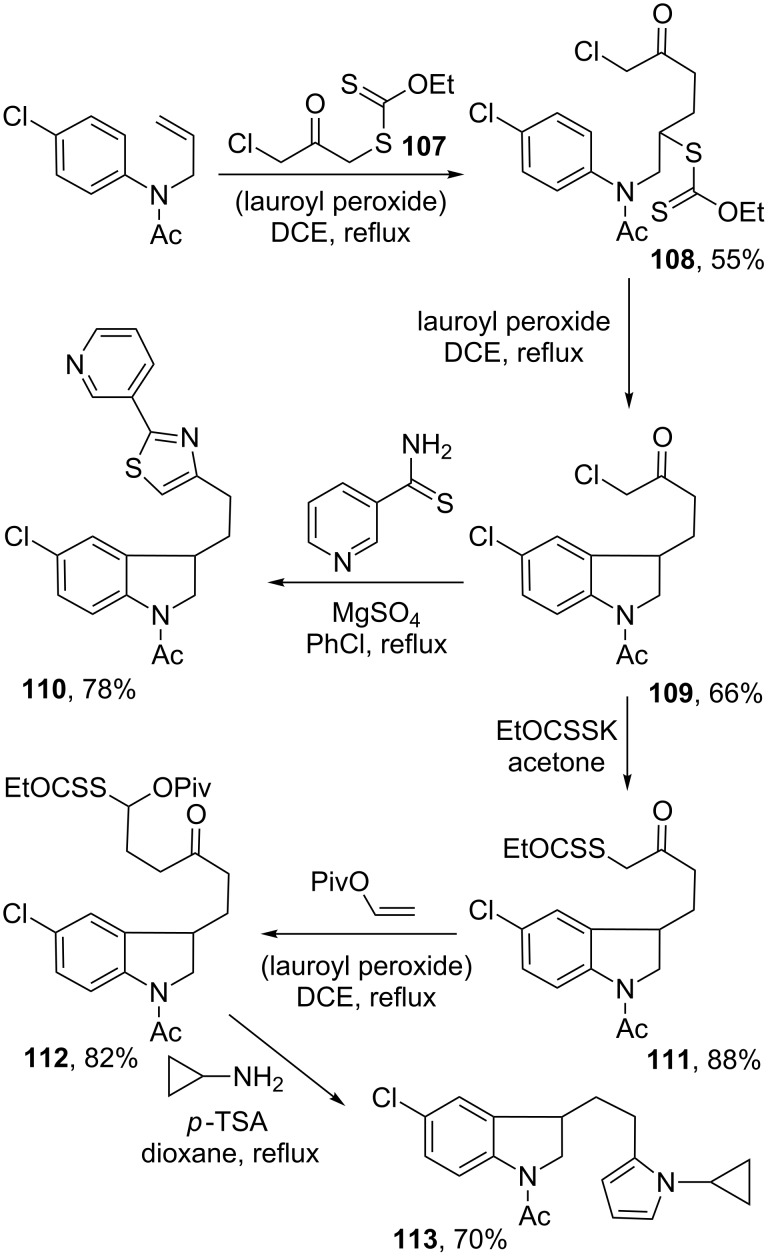
Multiple uses of a chloroacetonyl xanthate.

The second reagent is chloroacetonyl xanthate **107**, which, remarkably, is able to undergo clean radical additions despite the presence of the reactive chloroketone moiety, as demonstrated by its addition to *N*-allyl-*p*-chloroacetanilide to give **108** and cyclisation of the latter into indoline **109** [47]. Diverse otherwise inaccessible chloroketones become readily available. Haloketones in general are ideal precursors for the synthesis of numerous heteroaromatic derivatives. For example, Hantzsch condensation of indoline **109** with thionicotinamide furnishes thiazole **110** (Scheme 22). Also interesting is the possibility of substituting the chlorine in **109** with a xanthate salt to form a new xanthate **111** and performing a second radical addition to a different alkene such as vinyl pivalate. The adduct, **112** in this case, is the synthetic equivalent of a 1,4-keto-aldehyde and, in this capacity, can react with ammonia or primary amines, such as cyclopropylamine, to produce the corresponding pyrrole **113** by what may be viewed as a variation of the classical Paal–Knorr synthesis [47–48].

**Scheme 23:**
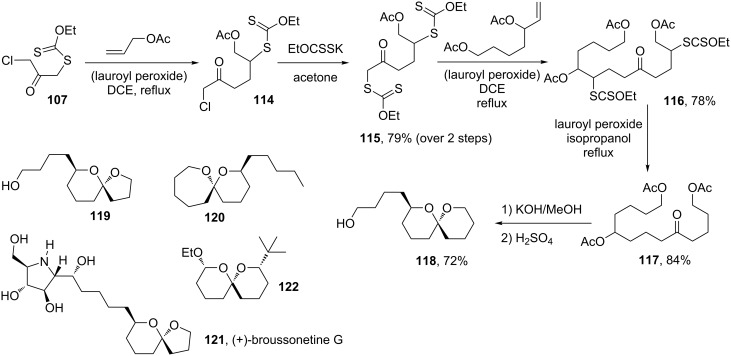
A convergent route to spiroketals.

Xanthate **107** is a highly versatile reagent, since it allows the attachment of differing chains on either side of the ketone group by two consecutive intermolecular radical additions to two different alkenes. An application of this property is a simple, yet general route to spiroketals and related derivatives, as shown by the sequence in Scheme 23 [49]. Thus, addition to allyl acetate gives the expected adduct **114**, where the chlorine can be readily displaced to provide dixanthate **115**. This compound reacts with a second alkene through the xanthate group that leads to the most stable radical, namely the one adjacent to the ketone, to furnish addition product **116**. Both xanthates can be reductively removed by treatment with stoichiometric amounts of peroxide in isopropanol as the solvent, and the resulting product **117** saponified and cyclised with acid into spiroketal **118**. By choosing a vinyl or a homoallyl ester as the alkene partner, spiroketals of various ring sizes can be easily constructed. Spiroketals **119** and **120** are two such examples. The former was used in the total enantioselective synthesis of (+)-broussonetine G (**121**) [50]. If one of the alkenes contains a masked aldehyde, a bis-spiroketal such as **122** may be accessed. Furthermore, placing a 1,2- or a 1,3-diol on one of the alkenes would in principle result in the formation of a cyclic ketal. Spiro and cyclic ketals are ubiquitous in pheromones and in marine natural products [51].

**Scheme 24:**
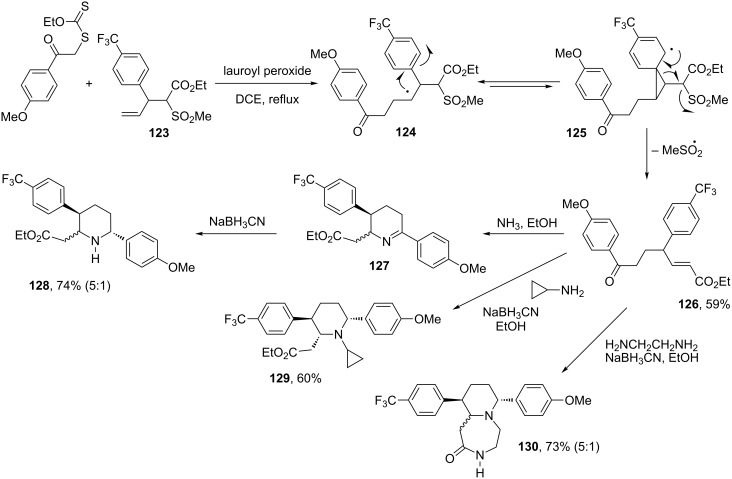
A modular approach to 3-arylpiperidines.

The comparatively long effective lifetime of radicals generated under the conditions of the xanthate transfer may be exploited to accomplish various difficult radical transformations. One particularly interesting process is the shifting of an aromatic ring through a radical Smiles rearrangement. In combination with the intermolecular xanthate addition, it becomes possible to rapidly assemble valuable precursors to 3-arylpiperidines. This strategy is illuminated by the synthetic outline in Scheme 24, where the intermediate radical **124** arising from addition to alkene **123** undergoes a 1,2-aryl shift via cyclopropane **125**, a process made irreversible by elimination of a methylsulfonyl radical to give ester **126** [52]. Two new C–C bonds are created, and the product now contains two electrophilic centres, a ketone and an unsaturated ester. Treatment with a primary amine or ammonia and in situ reduction of the intermediate imine **127** with sodium cyanoborohydride furnishes arylpiperidine **128** in good overall yield. Ammonia may be replaced with various primary amines, one example being cyclopropylamine, which furnishes piperidine **129** as one stereoisomer. In the case of 1,2-diaminoethane, a further cyclisation is observed leading to bicyclic piperidine **130**. All three components involved in this modular approach can be modified to provide a very broad diversity of structures.

### Radical vinylations and allylations

**Scheme 25:**
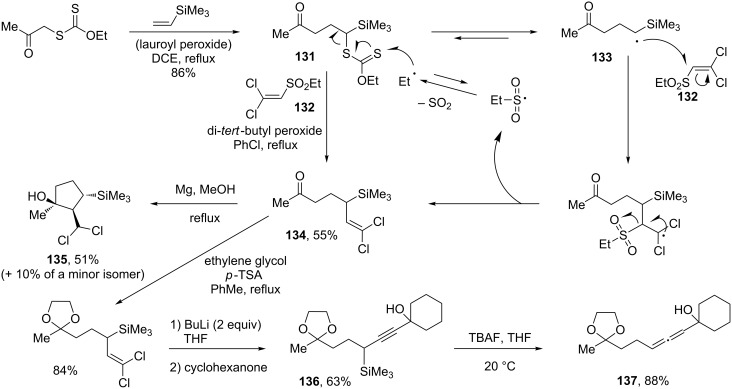
A convergent route to cyclopentanols and to functional allenes.

Xanthates may be incorporated into radical processes of various kinds. Vinylation and allylation reactions are particularly appealing, in view of the importance of such transformations in organic synthesis. A number of methods were developed to this end, with perhaps the most versatile relying on the chemistry of sulfones [53]. Aliphatic sulfonyl radicals are able to extrude sulfur dioxide, and the resulting carbon-centred radicals may be used in numerous ways. In particular, they can serve as chain-propagating agents for the xanthate-transfer process. An example is provided in Scheme 25 illustrating a rapid, convergent access to cyclopentanols and to functional allenes. Thus, the xanthate group in adduct **131**, derived from the radical addition to vinyl trimethylsilane, can be replaced by a dichlorovinyl motif through a second radical reaction with dichlorovinyl ethylsulfone (**132**) [54]. The ethylsulfonyl radical created in the addition–elimination process of intermediate radical **133** fragments to liberate sulfur dioxide and an ethyl radical that is capable of propagating the chain. The resulting product **134** possesses an interesting combination of functional groups. Upon treatment with magnesium turnings in methanol, this compound is converted into cyclopentanol **135** by capture of the intermediate ketyl radical by the dichlorovinyl group [55]. Alternatively, protection of the ketone and application of the Corey–Fuchs reaction followed by quenching of the acetylide with cyclohexanone furnishes propargyl silane **136**, a compound that is cleanly transformed into allene **137** upon exposure to tetrabutylammonium fluoride [54]. This approach represents perhaps the most versatile route to functional propargyl silanes. Indeed, a broad diversity of such structures becomes accessible by simply varying the starting xanthate and/or the ketone (or aldehyde) partner.

**Scheme 26:**
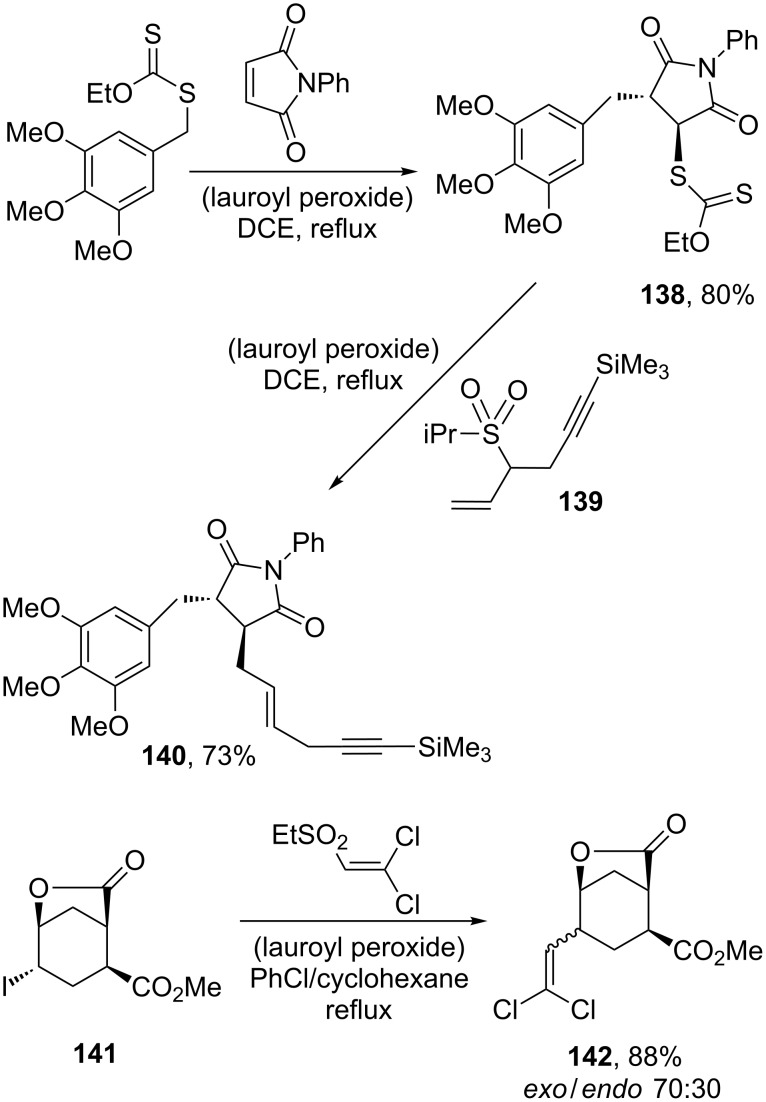
Allylation and vinylation of a xanthate and an iodide.

Allylations can be accomplished by applying the same concept, as shown by the sequence in Scheme 26 [56]. Again, complexity may be attained by combining the normal addition–transfer of a xanthate to an alkene with the allylation process to give fairly elaborate compounds, such as **140** obtained by reaction of adduct **138** with sulfone **139** [56]. It is interesting to note that both the vinylation and the allylation reactions are applicable to aliphatic iodides, as illustrated by the second transformation in Scheme 26 starting with iodide **141** and leading to dichlorovinyl lactone **142** [57]. It is interesting to note that while sp^2^–sp^2^ couplings are readily accomplished starting with vinylic or aromatic iodides using a variety of transition–metal catalysts, especially palladium-based complexes, sp^3^–sp^2^ couplings remain relatively rare, especially with secondary and tertiary aliphatic iodides. The present radical procedure therefore complements organometallic methods since it is most efficient with secondary and tertiary iodides, because the corresponding carbon radical is easiest to generate.

**Scheme 27:**
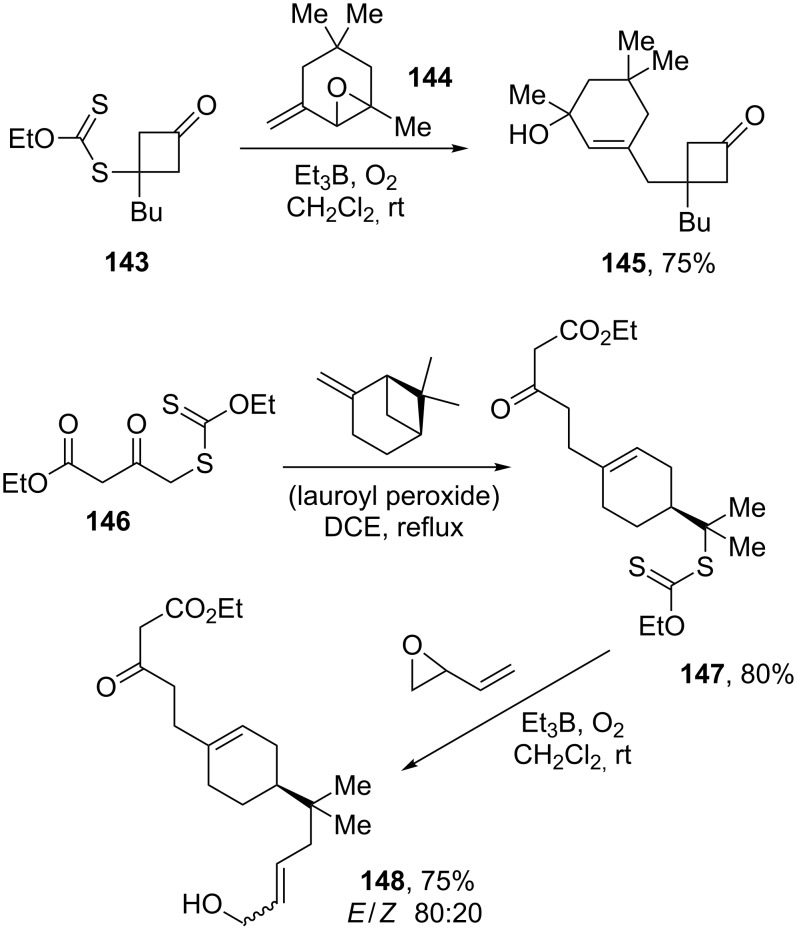
Vinyl epoxides as allylating agents.

Various radical allylating agents can effect the allylation of xanthates. These include substituted allyl diphenylphosphine oxides [58], vinyl epoxides [59], and allyl trimethylsilanes [60]. In the last case it is a two-step procedure. Two examples of the use of a vinyl epoxide as an allylating agent are displayed in Scheme 27 [59]. The first corresponds to the allylation of cyclobutyl xanthate **143** with vinyl epoxide **144** to yield compound **145**, while the second involves an addition–fragmentation of xanthate **146** on β-pinene to give xanthate **147**, which is then subjected to the allylation procedure with the simplest vinyl epoxide to furnish derivative **148**. It is interesting to note that the carbon–carbon bond formation takes place in ketoester xanthate **146** on the carbon bearing the least acidic hydrogens. Triethylborane, through its autoxidation, serves to initiate the process; it also quenches the intermediate alkoxy radical, liberating concomitantly an ethyl radical to propagate the chain.

**Scheme 28:**
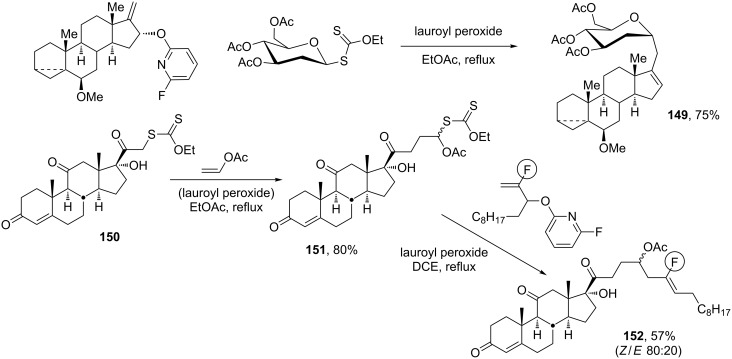
Radical allylations using allylic alcohol derivatives.

Except in the cleavage of epoxides, homolytic rupture of carbon–oxygen bonds is generally a difficult process. Radicals can thus be generated next to alcohols or esters without fear of β-elimination. This constitutes a tremendous synthetic asset and explains the popularity and importance of radical-based methods for the manipulation and modification of oxygen-rich compounds such as carbohydrates and cyclitols. It is, however, possible to transform an alcohol into a leaving group in the radical sense by converting it into a fluoropyridineoxy derivative by reaction of the alkoxide anion with inexpensive 2,6-difluoropyridine [61–65]. In this manner, any allylic alcohol becomes a potential radical allylating agent. This has proved to be a powerful route to complex alkenes, as indicated by the two examples in Scheme 28. The first represents a one-step synthesis of a steroid *C*-glycoside **149** [66], while the second illustrates a modular approach where the formation of alkene **152** is associated with the initial addition–transfer of cortisone derived xanthate **150** to vinyl acetate to provide intermediate xanthate **151** [65]. In this manner, a highly functionalised steroid containing a fluoro-substituted alkenyl side chain can be readily assembled. Both steroid products **149** and **152** would be exceedingly tedious to make by more conventional approaches.

The ability to generate an alkene by the homolytic cleavage of a C–O bond is a recent development that nevertheless holds much promise. It is open to numerous variations, in particular for the synthesis of highly functionalised ketones [64] and for the stereoselective formation of di- and tri-substituted alkenes [63].

### Generation of radicals by electron transfer from metallic nickel

**Scheme 29:**
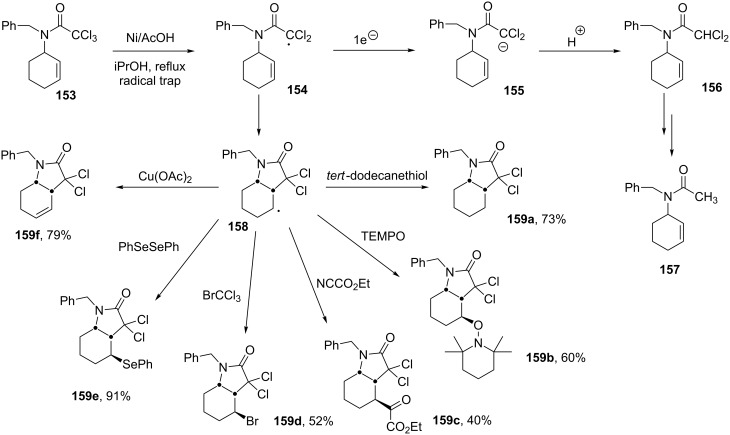
Synthesis of variously substituted lactams.

A final topic in this brief overview concerns the use of electron transfer from metallic nickel as a means for producing and capturing radicals from selected substrates. The underlying principle is quite simple. It hinges on the observation that the intermediate radical in the dissolving metal reduction of α-halocarbonyl derivatives could be captured by an internal alkene, if the second electron transfer is slow enough. For instance, in the dechlorination of trichloroacetamide **153** with the popular zinc/acetic acid combination, the second electron transfer leading to anion **155** is too rapid to allow interception of intermediate radical **154**. No cyclisation is thus observed under these conditions, which initially afford monodechlorinated product **156** and ultimately the completely reduced material **157** (Scheme 29). In contrast, replacing zinc with plain nickel powder and diminishing the acidity of the medium by addition of a cosolvent allows the cyclisation to proceed. The resulting secondary radical **158** is not sufficiently electrophilic in character to be reduced by the metal and can be trapped by various external reagents, as exemplified by the rich array of lactams **159a**–**f** [67]. In the case of ketoester **159c**, the reaction was carried out at room temperature [68]. The formation of unsaturated lactam **159f** is particularly interesting, since it involves the use of a mild oxidant (cupric acetate) in a reducing medium [69]. Another remarkable feature is that the nickel/acetic acid reducing system is capable of cleanly distinguishing between the starting trichloroacetamide and the dichlorolactam product. They have relatively close reduction potentials, yet the latter is reduced much more slowly under these conditions.

**Scheme 30:**
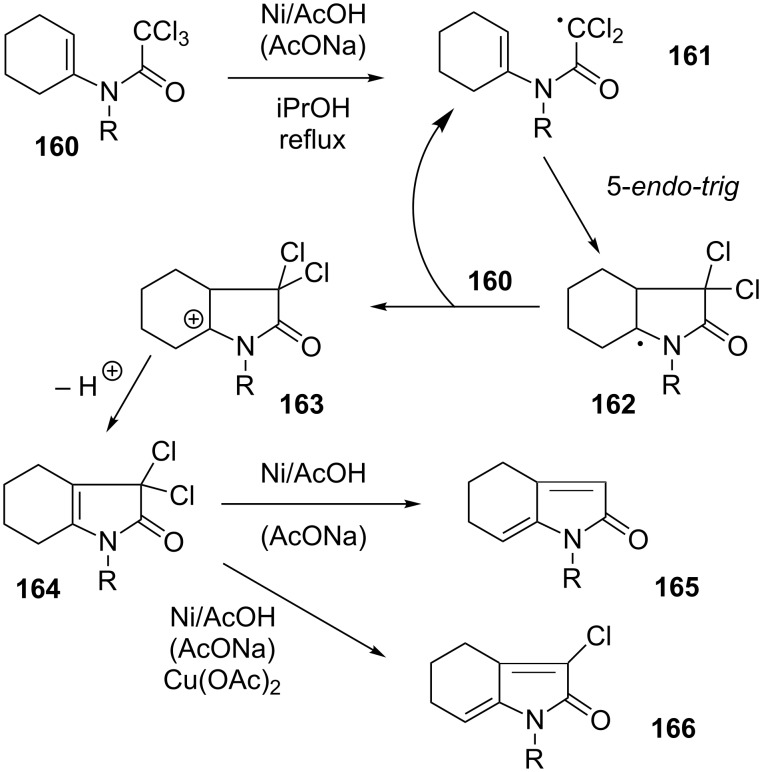
Nickel-mediated synthesis of unsaturated lactams.

A further synthetically valuable observation is the behaviour of ene-trichloroacetamides such as **160** (Scheme 30) [70]. In this system, the intermediate radical **161** undergoes a 5-*endo* cyclisation into the easily oxidised tertiary radical **162**, which is then logically converted into cationic species **163** by electron transfer to the starting trichloroacetamide **160**. Loss of a proton finally provides unsaturated dichlorolactam **164**. This compound cannot be isolated because the chlorines are now allylic and therefore easier to reduce off than those of the starting material. Thus, a second chlorine atom is lost through reduction and the last is simply eliminated as chloride to give diene **165** as the final product. In the presence of cupric acetate additive, which somehow slows down the reduction, the elimination of chloride is faster than reduction, and it is chlorodiene **166** that is ultimately formed [70]. Since HCl is formally produced in these transformations, the addition of sodium acetate to buffer the medium is often beneficial.

**Scheme 31:**
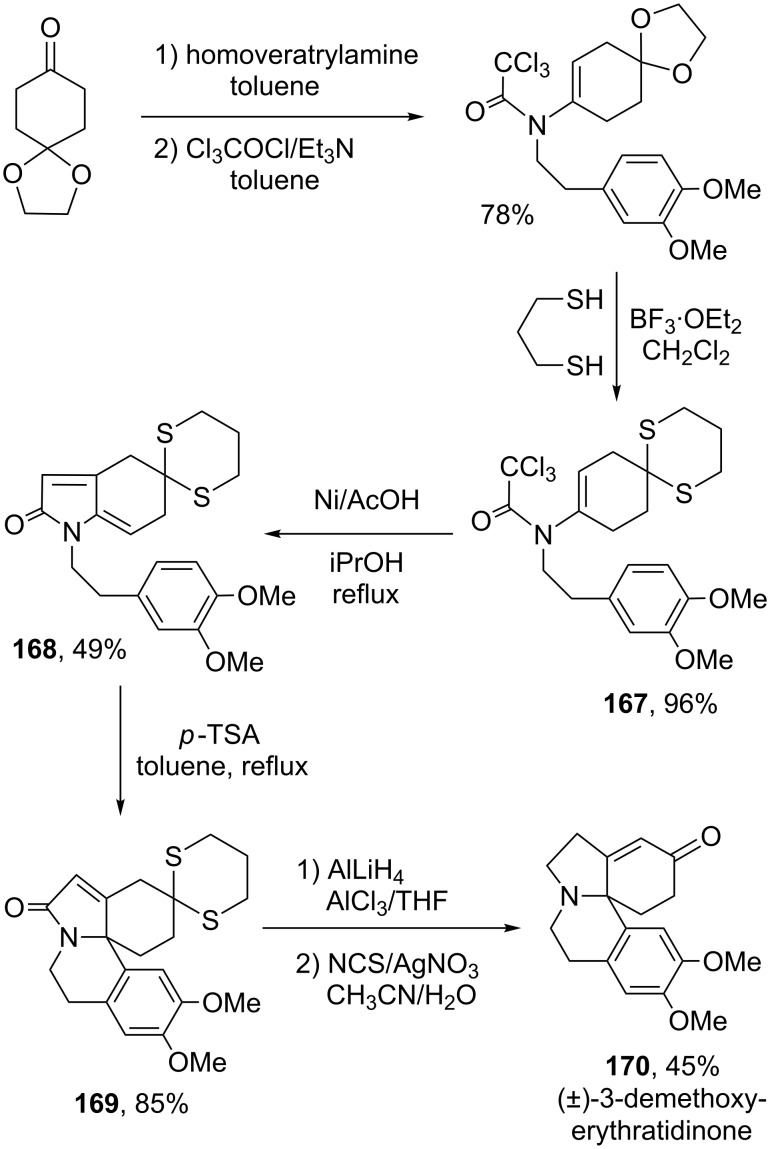
Total synthesis of (±)-3-demethoxy-erythratidinone.

The easy access to unsaturated lactams such as **165** and **166** may be exploited to construct complex structures by subsequent application of radical, ionic, or organometallic transformations. This is illustrated by the short, six-step total synthesis of (±)-3-demethoxy-erythratidinone (**170**) displayed in Scheme 31 [71]. In this approach, trichloroacetenamide **167**, trivially obtained in two steps from monoprotected cyclohexanedione, is subjected to the cyclisation sequence to give diene **168**, which smoothly undergoes an intramolecular Friedel–Crafts reaction to generate the last ring in intermediate **169**. Finally, reduction of the lactam and unmasking of the ketone causes the migration of the olefin to complete the synthesis of the target structure **170**.

**Scheme 32:**
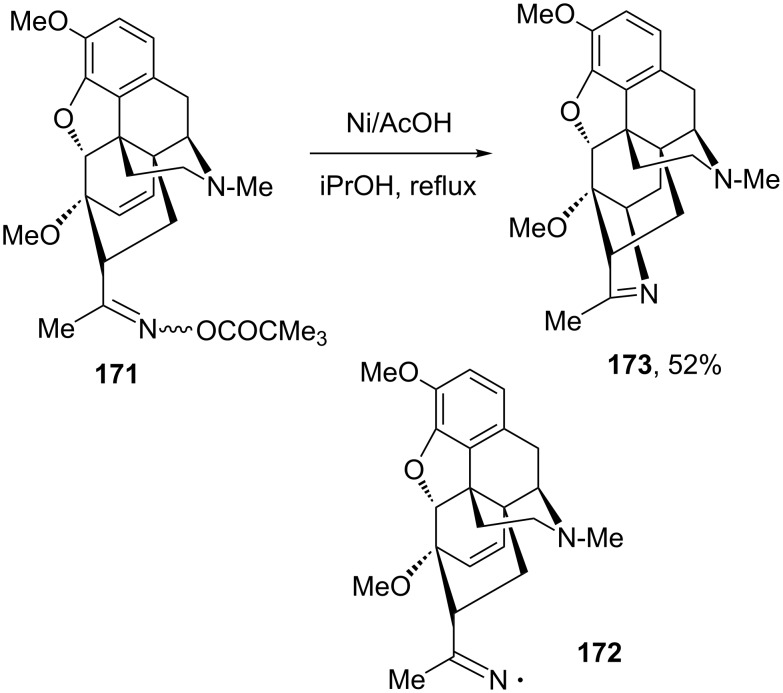
Generation and capture of an iminyl radical from an oxime ester.

The generation of radicals using nickel powder in combination with acetic acid can be extended to the production of iminyls by reduction of oxime esters [72–73]. This variant allows an easy access to pyrrolenines as underscored by the synthesis of complex structure **173** from thebain-derived oxime pivalate **171** via iminyl radical **172** (Scheme 32) [73]. In this transformation, the solvent, 2-propanol, acts as the final hydrogen atom donor to quench the carbon radical formed upon cyclisation of the iminyl.

## Conclusion

The preceding examples showcase some of the possibilities for creating complexity by using radical processes we have developed over the years. Our research is curiosity driven, guided by a search for novelty and a thirst for understanding reaction mechanisms. Constructing complex molecular architectures is only incidental to our work, mostly a consequence of our exploration of the scope and limitations of our methods. The degenerative radical addition–transfer of xanthates is by far the most powerful radical chemistry that we have been able to discover, for it allows efficient intermolecular carbon–carbon bond formation starting with unactivated alkenes under very mild experimental conditions. Various functional groups can therefore be brought together and made to react in an infinite number of ways. Because of its convergence and tolerance, the xanthate technology offers numerous possibilities for the rapid assembly of well-decorated, intricate carbon frameworks. Much work has been done, but much more remains to be done in this never-ending quest, which will hopefully continue to take us to unexpected venues.

## References

[1] Tietze L F, Brasche G, Gericke K M (2006). Domino Reactions in Organic Synthesis.

[2] Renaud P, Sibi M P (2001). Radicals in Organic Synthesis.

[3] Zard S Z (2003). Radical Reactions in Organic Synthesis.

[4] Gansäuer A (2006). Radicals in Synthesis II.

[5] Chatgilialoglu C, Studer A (2012). Encyclopedia of Radicals in Chemistry, Biology and Materials.

[6] Zard S Z (2008). Chem Soc Rev.

[7] Boivin J, Callier-Dublanchet A-C, Quiclet-Sire B, Schiano A-M, Zard S Z (1995). Tetrahedron.

[8] Cassayre J, Zard S Z (1999). J Am Chem Soc.

[9] Cassayre J, Zard S Z (2001). J Organomet Chem.

[10] Brummond K M, Kent J L (2000). Tetrahedron.

[11] Blanco-Urgoiti J, Añorbe L, Pérez-Serrano L, Domínguez G, Pérez-Castells J (2004). Chem Soc Rev.

[12] Cassayre J, Gagosz F, Zard S Z (2002). Angew Chem, Int Ed.

[13] Callier-Dublanchet A-C, Cassayre J, Gagosz F, Quiclet-Sire B, Sharp L A, Zard S Z (2008). Tetrahedron.

[14] Biéchy A, Hachisu S, Quiclet-Sire B, Ricard L, Zard S Z (2008). Angew Chem, Int Ed.

[15] Biéchy A, Hachisu S, Quiclet-Sire B, Ricard L, Zard S Z (2009). Tetrahedron.

[16] Boivin J, Fouquet E, Schiano A-M, Zard S Z (1994). Tetrahedron.

[17] Zard S Z (1996). Synlett.

[18] Barton D H R, da Silva E, Zard S Z (1988). J Chem Soc, Chem Commun.

[19] Chatterjee A K, Choi T-L, Sanders D P, Grubbs R H (2003). J Am Chem Soc.

[20] Delduc P, Tailhan C, Zard S Z (1988). J Chem Soc, Chem Commun.

[21] Quiclet-Sire B, Zard S Z (2011). Pure Appl Chem.

[22] Quiclet-Sire B, Zard S Z (2010). Heterocycles.

[23] Chatgilialoglu C (1995). Chem Rev.

[24] Cooper R D G, José F L (1972). J Am Chem Soc.

[25] Quiclet-Sire B, Revol G, Zard S Z (2010). Tetrahedron.

[26] Boiteau L, Boivin J, Liard A, Quiclet-Sire B, Zard S Z (1998). Angew Chem, Int Ed.

[27] Cordero-Vargas A, Quiclet-Sire B, Zard S Z (2005). Org Biomol Chem.

[28] Legrand N, Quiclet-Sire B, Zard S Z (2000). Tetrahedron Lett.

[29] Petit L, Zard S Z (2010). Chem Commun.

[30] Bacqué E, El Qacemi M, Zard S Z (2004). Org Lett.

[31] Biéchy A, Zard S Z (2009). Org Lett.

[32] Boutillier P, Quiclet-Sire B, Zafar S N, Zard S Z (2010). Tetrahedron: Asymmetry.

[33] Quiclet-Sire B, Sortais B, Zard S Z (2002). Synlett.

[34] Quiclet-Sire B, Woollaston D, Zard S Z (2008). Tetrahedron.

[35] Binot G, Zard S Z (2003). Tetrahedron Lett.

[36] Cholleton N, Gauthier-Gillaizeau I, Six Y, Zard S Z (2000). Chem Commun.

[37] Martín Castro A M (2004). Chem Rev.

[38] Briggs M E, El Qacemi M, Kalaï C, Zard S Z (2004). Tetrahedron Lett.

[39] Revol G, Fuchs C, Zard S Z (2012). Can J Chem.

[40] Denieul M-P, Quiclet-Sire B, Zard S Z (1996). Tetrahedron Lett.

[41] Alameda-Angulo C, Quiclet-Sire B, Zard S Z (2006). Tetrahedron Lett.

[42] Hook J M, Mander L N (1986). Nat Prod Rep.

[43] Braun M-G (2011). Applications de la chimie radicalaire des xanthates.

[44] Sortais B (2002). Nouvelles synthèses radicalaires d’indanes, de dérivés d’hydrazines et d‘hydroxylamines. Nouvel accès aux alcaloïdes de l’ergoline et synthèse radicalaire de la mélatonine.

[45] Liu Z, Qin L, Zard S Z (2012). Org Lett.

[46] Corbet M, de Greef M, Zard S Z (2008). Org Lett.

[47] Bergeot O, Corsi C, El Qacemi M, Zard S Z (2006). Org Biomol Chem.

[48] Quiclet-Sire B, Quintero L, Sanchez-Jimenez G, Zard S Z (2003). Synlett.

[49] de Greef M, Zard S Z (2007). Org Lett.

[50] Trost B M, Horne D B, Woltering M J (2003). Angew Chem, Int Ed.

[51] Perron F, Albizati K F (1989). Chem Rev.

[52] Georghe A, Quiclet-Sire B, Vila X, Zard S Z (2007). Tetrahedron.

[53] Bertrand F, Le Guyader F, Liguori L, Ouvry G, Quiclet-Sire B, Seguin S, Zard S Z (2001). Acad Sci, C R Ser II.

[54] Li Z, Zard S Z (2009). Org Lett.

[55] Li Z, Zard S Z (2009). Tetrahedron Lett.

[56] Charrier N, Zard S Z (2008). Angew Chem, Int Ed.

[57] Bertrand F, Quiclet-Sire B, Zard S Z (1999). Angew Chem, Int Ed.

[58] Ouvry G, Quiclet-Sire B, Zard S Z (2006). Angew Chem, Int Ed.

[59] Charrier N, Gravestock D, Zard S Z (2006). Angew Chem, Int Ed.

[60] Briggs M E, Zard S Z (2005). Synlett.

[61] Charrier N, Quiclet-Sire B, Zard S Z (2008). J Am Chem Soc.

[62] Brioche J, Michalak M, Quiclet-Sire B, Zard S Z (2011). Org Lett.

[63] Braun M-G, Quiclet-Sire B, Zard S Z (2011). J Am Chem Soc.

[64] Debien L, Quiclet-Sire B, Zard S Z (2011). Org Lett.

[65] Debien L, Quiclet-Sire B, Zard S Z (2012). Org Lett.

[R66] 66Kosnik, W.; Zard, S. Z. unpublished observations.

[67] Boivin J, Yousfi M, Zard S Z (1994). Tetrahedron Lett.

[68] Yousfi M (1995). Etude de nouvelles réactions radicalaires induites par le nickel en poudre.

[69] Cassayre J, Dauge D, Zard S Z (2000). Synlett.

[70] Cassayre J, Quiclet-Sire B, Saunier J-B, Zard S Z (1998). Tetrahedron.

[71] Cassayre J, Quiclet-Sire B, Saunier J-B, Zard S Z (1998). Tetrahedron Lett.

[72] Boivin J, Schiano A-M, Zard S Z (1992). Tetrahedron Lett.

[73] Boivin J, Schiano A-M, Zard S Z, Zhang H (1999). Tetrahedron Lett.

